# Towards a standardized program of transitional care for adolescents with juvenile idiopathic arthritis for Turkey: a national survey study

**DOI:** 10.1186/s12969-023-00943-3

**Published:** 2024-01-02

**Authors:** Betül Sözeri, Nihal Şahin, Ceyhun Açarı, Pinar Ozge Avar Aydın, Ozge Baba, Esra Bağlan, Sevcan Bakkaloğlu, Sibel Bakırcı, Yelda Bilginer, Burcu Yücel Bozkaya, Şengül Çağlayan, Mustafa Çakan, Figen Çakmak, Taner Coşkuner, Ferhat Demir, Fatma Gül Demirkan, Şeyda Doğantan, Hatice Adıgüzel Dündar, Emine Duygu Ersözlü, Sercan Gücenmez, Oğuz Gürler, Rana İşgüder, Adem Küçük, Mukaddes Kalyoncu, Levent Kılıç, Sara Şebnem Kılıç, Hakan Kısaoğlu, Ayşenur Paç Kısaarslan, Zehra Kızıldağ, Duygu Kurtuluş, Semanur Özdel, Kübra Öztürk, Pelin Şenol, Ayşe Tanatar, Sema Nur Taşkın, Fatma Tuncer Kuru, Serkan Türkuçar, Kadir Ulu, Erbil Ünsal, Ayten Yazıcı, Deniz Gezgin Yıldırım, Selçuk Yüksel, Özgür Kasapçopur, Seza Özen, Nuray Aktay Ayaz, Hafize Emine Sönmez

**Affiliations:** 1 Department of Pediatric Rheumatology, University of Health Sciences, Umraniye Research and Training Hospital, Istanbul, Turkey; 2 Department of Pediatric Rheumatology, Faculty of Medicine, Kocaeli University, Kocaeli, Turkey; 3 Department of Pediatric Rheumatology, Faculty of Medicine, Inönü University, Malatya, Turkey; 4 Department of Pediatric Rheumatology, Tekirdağ State Hospital, Tekirdağ, Turkey; 5 Department of Pediatric Rheumatology, Karadeniz Technical University Faculty of Medicine, Trabzon, Turkey; 6 Department of Pediatric Rheumatology, Etlik State Hospital, Ankara, Turkey; 7 Department of Pediatric Rheumatology, Faculty of Medicine, Gazi University, Ankara, Turkey; 8 Department of Rheumatology, Antalya State Hospital, Antalya, Turkey; 9 Department of Pediatric Rheumatology, Faculty of Medicine, Hacettepe University, Ankara, Turkey; 10 Department of Pediatric Rheumatology, Samsun Research and Training Hospital, Samsun, Turkey; 11 Department of Pediatric Rheumatology, Zeynep Kamil Research and Training Hospital, Istanbul, Turkey; 12 Department of Pediatric Rheumatology, Başakşehir Çam and Sakura State Hospital, Istanbul, Turkey; 13 Department of Pediatric Rheumatology, Acıbadem Hospital, Istanbul, Turkey; 14 Department of Pediatric Rheumatology, Istanbul Faculty of Medicine, Istanbul University, Istanbul, Turkey; 15 Department of Pediatric Rheumatology, Mersin City Hospital, Mersin, Turkey; 16 Department of Pediatric Rheumatology, Behçet Uz Research and Training Hospital, Izmir, Turkey; 17 Department of Rheumatology, Adana City Research and Training Hospital, Adana, Turkey; 18 Department of Rheumatology, Izmir Atatürk Research and Training Hospital, Izmir, Turkey; 19 Department of Rheumatology, Medikal Park Hospital, Samsun, Turkey; 20 Department of Pediatric Rheumatology, Faculty of Medicine, Dokuz Eylül University, Izmir, Turkey; 21 Department of Rheumatology, Faculty of Medicine, Konya Selçuk University, Konya, Turkey; 22 Department of Rheumatology, Faculty of Medicine, Hacettepe University, Ankara, Turkey; 23 Department of Pediatric Rheumatology, Faculty of Medicine, Uludağ University, Bursa, Turkey; 24 Department of Pediatric Rheumatology, Kayseri City Hospital, Kayseri, Turkey; 25 Department of Pediatric Rheumatology, Faculty of Medicine, Erciyes University, Kayseri, Turkey; 26 Department of Physical Therapy and Rehabilitation, Umraniye Training and Research Hospital, Istanbul, Turkey; 27 Department of Pediatric Rheumatology, Göztepe Research and Training Hospital, Istanbul, Turkey; 28 Department of Pediatric Rheumatology, Eskişehir City Hospital, Eskişehir, Turkey; 29 Department of Rheumatology, Osmaniye State Hospital, Osmaniye, Turkey; 30 Department of Pediatric Rheumatology, Faculty of Medicine, Pamukkale University, Denizli, Turkey; 31 Department of Rheumatology, Faculty of Medicine, Kocaeli University, Kocaeli, Turkey; 32 Department of Pediatric Rheumatology, Faculty of Medicine, Onsekiz Mart University, Çanakkele, Turkey; 33 Department of Pediatric Rheumatology, Istanbul University-Cerrahpaşa, Cerrahpaşa Medical School, Istanbul, Turkey

**Keywords:** Adolescent, Arthritis, Juvenile, Chronic disease, Transition to adult care

## Abstract

**Background:**

Juvenile idiopathic arthritis (JIA) is a prevalent childhood chronic arthritis, often persisting into adulthood. Effective transitional care becomes crucial as these patients transition from pediatric to adult healthcare systems. Despite the concept of transitional care being recognized, its real-world implementation remains inadequately explored. This study aims to evaluate the thoughts and practices of healthcare providers regarding transitional care for JIA patients.

**Methods:**

A cross-sectional survey was conducted among pediatric and adult rheumatologists in Turkey. Based on the American Academy of Pediatrics’ six core elements of transitional care, the survey included 86 questions. The respondents’ demographic data, attitudes towards transitional care, and practical implementation were assessed.

**Results:**

The survey included 48 rheumatologists, with 43.7% having a transition clinic. The main barriers to establishing transition programs were the absence of adult rheumatologists, lack of time, and financial constraints. Only 23.8% had a multidisciplinary team for transition care. Participants agreed on the importance of coordination and cooperation between pediatric and adult healthcare services. The timing of the transition process varied, with no consensus on when to initiate or complete it. Participants advocated for validated questionnaires adapted to local conditions to assess transition readiness.

**Conclusions:**

The study sheds light on the challenges and perspectives surrounding transitional care for JIA patients in Turkey. Despite recognized needs and intentions, practical implementation remains limited due to various barriers. Cultural factors and resource constraints affect the transition process. While acknowledging the existing shortcomings, the research serves as a ground for further efforts to improve transitional care and ensure better outcomes for JIA patients transitioning into adulthood.

**Supplementary Information:**

The online version contains supplementary material available at 10.1186/s12969-023-00943-3.

## Background

Juvenile idiopathic arthritis (JIA) is the most prevalent chronic arthritis in childhood, affecting approximately 16 to 150 per 100,000 children [1]. With advancements in the treatment of pediatric rheumatic diseases, a growing number of children now reaching the age of 18 and require a shift from pediatric to adult healthcare systems [2]. Despite some JIA patients achieving remission, a significant proportion, ranging from 30–60%, continue to experience active disease during adulthood [3–6]. Therefore, clinicians must ensure the continuation of the health services of patients with JIA in adulthood. Consequently, the concept of transitional care has emerged as a burgeoning area of research and practice within the field of pediatric rheumatology. The Society of Adolescent Medicine has described the transition as “a purposeful and planned process for adolescents and young adults (AYAs) with a chronic disease while moving from child-centered to adult-oriented health care systems” [7, 8]. Despite the clear conceptual definition of transition, there is insufficient data on its functioning in real life. A recent survey conducted among European pediatric rheumatologists revealed that less than one-third of respondents reported having a written transition policy [9]. Data for developing countries are much scarcer. Only one survey from a developing country, Brazil, reported that 13% of pediatric rheumatology centers had a well-established transition program [10]. Herein, we aimed to evaluate the thoughts of healthcare providers on transitional care and how transitional care works in real life.

## Methods

A cross-sectional survey was conducted to evaluate the thoughts of healthcare providers on transitional care and how transitional care works in real life. The open online survey which was available between 15th and 30th July 2023 was designed by using the Google Forms software (Google Forms, Albuquerque, New Mexico, USA). The survey included anonymous questionnaires and was sent to the members of the Pediatric and Adult Rheumatology associations via e-mail. The survey consisted of 86 questions (Supplementary material). The questions were designed according to the American Academy of Pediatrics’ six core elements: (1) transition policy, (2) tracking and monitoring, (3) transition readiness, (4) transition planning, (5) transfer of care, and (6) transition completion [11, 12]. A comprehensive assessment was conducted to measure the level of belief of pediatric and adult rheumatologists and how much they used the items included in the six basic elements in real-life practice. The targeted population included both pediatric and adult rheumatologists practicing in different parts of Turkey. The Ethics Committee approved the study protocol and adhered to the principles of the Declaration of Helsinki (2023/297).

### Statistical analysis

All statistical analyses were performed using SPSS for Windows v. 22.0 (IBM, Armonk, NY, United States). The Kolmogorov–Smirnov, and Shapiro– Wilk tests were used to assess the normality assumption. Numeric variables were presented as median ± (minimum-maximum). Categorical variables were summarized as counts (percentages).

## Results

### Demographic data of respondents

A total of 48 rheumatologists participated in this study. Of them, 39 were pediatric rheumatologists, and 9 were adult rheumatologists. Twenty-four of the participants were working in a public hospital, while 22 were in a university hospital and 2 were in a private hospital. The median duration of working in the field of rheumatology was 4 (2–35) years. The respondents stated that patients aged 14–22 years make up approximately one-third of their practice.

### Transition policy

This section questioned whether there was a standard transition policy or not. Twenty-seven (56.3%) participants stated that they did not have a transition clinic, while 21 (43.7%) reported that they had a transition clinic. A transition clinic was defined as a standard clinic working in cooperation with at least one pediatric rheumatologist, and one adult rheumatologist. The main barriers to developing a transition program were the absence of adult rheumatologists in the center, lack of time, and the current health system not financing the transition outpatient clinic (Table 1).

**Table 1:** The barriers experienced by participants in the transition process

The barriers experienced in the transition	Response percentage
***The reasons for the inability to generate the transition program in centers (n=27)***	
Lack of an adult rheumatology clinic in the center	51.9%
Lack of time	40.7%
Social insurance-related appointment and registration problems	29.6%
Financial inadequacy	11.1%
***The reasons for not deciding on timing transition steps with caregivers (n=37)***	
Lack of time	64.9%
Negative attitudes of patients and parents about this issue	51.4%
The existence of a transitional program that is not suitable for this subject	24.3%
***The reasons for the inability to choose a transfer center in cooperation between patients, parents and clinicians (n=18)***	
Transferring by pediatric rheumatologists only to adult rheumatologists in their centers	55.6%
Parents prefer an adult rheumatology center that aligns with their own conditions	33.3%
Transferring to a specific adult rheumatology center not allowed by the national health appointment system	27.8%

Respondents were able to select more than one reason for items.

All participants agreed on the necessity of a transitional policy developed with youth and families including the whole steps in the transition process. Out of the 21 respondents who stated that they had a transition outpatient clinic, only 16 (76.1%) reported having a standardized transitional policy and program, and only 5 (23.8%) had a multidisciplinary team for the transition. Transition teams were mainly led by pediatric rheumatologists, adult rheumatologists, physiotherapists, and nurses. The remaining 16 participants who did not have a multidisciplinary team attributed this to the lack of employees.

Fourteen (29.1%) respondents stated that a person coordinated the transition period in their center. The designated team member as a transition coordinator was a pediatric rheumatologist in almost all centers except one. Although all participants underlined the necessity of good cooperation between pediatric and adult health services, only 2 of them reported that the communication between pediatric and adult health services was sufficient.

Although 40 (83.3%) respondents stated that the transfer center should be selected in cooperation between patients, parents, and clinicians, 18 of them reported that they could not jointly select the transfer center because of specific reasons depicted in Table 1.

### Tracking and monitoring

This section questioned whether there was a criterion for identifying transition-aged youth or a standard recording process or not. Most respondents (*n* = 30, 62.5%) stated that the patient’s age was the most important factor for timing the transition. Thirty-one of them stated that preparation should start between 16 and 18 years, while 15 of them suggested between 14 and 16 years, and 2 of them proposed between 12 and 14 years (Table 2).

**Table 2:** The rate of agreement among participants on the age at which the transition starts, transfer of care, readiness, and completion of the transition

Timing of transition steps	The ratio of agreement
Preparation of the transition process should start at 16–18 years old	64.6%
at 14–16 years old	31.2%
at 12–14 years old	4.2%
The first talk with the patient about the transition should be made at 12–14 years old.	62.5%
The transition program planning should start between 16 and 18 years.	95.8%
The transition visits should be made between 18 and 20 years.	60%
The transition process should be completed between 20–24 years.	52.7%
**Statements for the transition readiness**	
The timing of all transition steps until the age of 18 should be cleared during the transition planning step.	54.2%
Parents’ new changing roles in the transition should be discussed.	75%
The patient should be encouraged to answer questions about their illness, treatment, pain, education, and activities during visits after the age of 12.	70%
**Statements for the transfer of care**	
During the transfer visit, the logbook of the transition period and the epicrisis should be submitted to the adult rheumatology department.	95.8%
The second visit should be made together with pediatric and adult rheumatologists in the adult rheumatology outpatient clinic.	75.1%
The feedback of parents or patients on adult rheumatology care should be taken by pediatric rheumatology at the first or second transfer visit.	87.5%
To ensure that the patient continues with adult rheumatology care until the age of 24, they should be seen annually in the pediatric rheumatology clinic after the transfer.	16.1%
**Statements for the transition completion**	
The last visit should only be conducted in the adult rheumatology clinic.	43.8%
Make sure that the patient schedules follow-up appointments before concluding the transition process.	75%
The patient should be seen alone in the visits after the transfer visit.^a^	43.8%

^a^This question was answered only by adult rheumatologists.

Furthermore, 35 (72.9%) respondents addressed the necessity of recording the data of the transition process, while only 15 of them had a regular registration system for the transition process. The main reasons were a lack of time and financial problems.

### Transition readiness

This section questioned whether there were regular transition readiness assessments or not. The readiness for transition may be evaluated by using standard surveys or by measuring the patient’s level of knowledge about their own disease and medications.

Although 30 (62.5%) respondents stated that the first talk with patients about the transition should be made between 12 and 14 years old and that their readiness for the transition should be tested, only 11 (22.9%) of the respondents reported that they tested the patients’ readiness before starting the transition outpatient clinic. The remaining 37 respondents stated that they could not assess the readiness of patients due to a lack of time and auxiliary staff.

Forty (83.3%) respondents agreed that transfer readiness should be assessed through validated questionnaires such as TRAQ and transition Q, and 26 (54.2%) respondents thought these questionnaires should be rearranged according to cultural, sociodemographic, and health insurance conditions.

While more than two-thirds of respondents (*n* = 39, 81.2%) believe that parents should enroll in the timing of transition steps, only 11 of them (22.9%) reported that parents enroll while timing the transition steps in their clinics. The most important factor preventing deciding on timing transition steps with caregivers is the lack of time and their attitudes towards this issue (Table 1). In other opinions questioned, the ratio of agreement was 50–75% (Table 2).

#### Transition planning

Planning a proper transition should include assessment of readiness, goals of the youth’s and their prioritized actions, and an emergency care plan. In this section participants were questioned about their thoughts on these issues.

Almost all respondents (*n* = 44, 91.6%) advised that patients should be questioned about their knowledge of their diseases and drugs. Only 19 (39.5%) respondents were informed about career choices related to illness. The rest of the respondents reported that they avoid discussing career choices due to a lack of time and the attitudes of patients and parents on this issue.

Although most of the respondents (*n* = 44, 91.6%) agreed that they should inform patients about sexuality and the harms of smoking, alcohol, and narcotics, clinicians refused to talk about these issues due to sexuality being taboo in our country and parents’ reactions to the usage of tobacco, alcohol, and narcotics.

Thirty-one responders stated that they involve parents in the decision when scheduling transition steps in their clinics.

### Transfer of care

This section examined the attitudes and approaches of responders regarding the time of the initial transfer visit, submission of medical summaries, and scheduling an adult clinical appointment.

Twenty-nine (60%) respondents stated that transition visits should be made between 18 and 20 years. However, 33 (68.8%) participants reported that the transition visit was made at the age of 18, 11 (22.9%) between the ages of 18–20, and 4 (8.3%) between the ages of 20–22.

Almost all responders agreed the necessity of submitting a medical summary to the adult rheumatology department during transfer visit. Furthermore, almost all confirmed that they submitted the epicrisis during transfer visit.

Of the respondents, 66.6% (*n* = 32) agreed that the transfer should be timed at stable disease. Fifteen respondents cited being unable to transfer at stable disease because some patients needed treatments not yet approved for pediatrics or the pediatric rheumatologists. Forty (83.3%) respondents agreed that the transfer visit should be conducted in an environment suitable for AYAs, but only 12 (25%) had the appropriate setting. While 43 (89.6%) respondents stated that at least one transfer visit should be done together with adult rheumatology and pediatric rheumatology, 17 (35.4%) had practiced that way. Other opinions questioned regarding the transfer of care are given in Table 2. Up to 85% responders confirmed the necessity of feedback about the transfer visit. However, only one-third of responders stated that they got feedback after the transfer visit.

### Transition completion

All questions about the transition completion step were answered by all participants except one question, as stated in Table 2. About half of the respondents (52.7%, *n* = 25) agreed that the transition process should be completed in 20–24 years. When all respondents were asked at what age concluded the transition process in practical terms, they stated 31.3% had completed the transition at 18, 37.5% between 18 and 20, and another 31.3% between 20 and 24. These findings do not constitute conclusive results derived from patient data. Forty-two (87.5%) thought patients should have a clear plan for education, professional life, and disease management before completing the transition.

## Discussion

In Turkey, before the pediatric rheumatology department was officially declared as a division of pediatrics, children with rheumatic diseases had received care in various divisions of pediatrics (https://cocukromatoloji.org). Since then, the number of pediatric rheumatology clinics and the patient population under their care has steadily grown. The estimated prevalence of JIA was reported to be 0.032% in our region [13]. Following periodic fever syndromes, JIA emerges as the primary concern in the field of pediatric rheumatology in our nation [14]. Unfortunately, a substantial portion of these patients continue to experience active disease in adulthood [3–6]. Therefore, establishing a proper transfer program for these patients has become inevitable. In this survey study, we revealed insights into the current state of transition care for JIA and presented the perspectives of both pediatric and adult rheumatologists regarding the transition process within our country.

Respondents to this survey mainly consisted of pediatric rheumatologists, and half of them have been employed within a public healthcare institution, while less than half of all participants declared that they had a transition clinic. Only 16 participants reported that they enrolled in a standard transition program, and among them, a smaller proportion (*n* = 5, 23.8%) provided transitional care services with a multidisciplinary team. While the prevalence of a multidisciplinary transition program in our country was notably lower than that in Canada [15], a developed country, it exhibited a similarity to the situation in Brazil [10], a developing country like ours, that may be attributed to disparities between developing and developed countries. Unexpectedly, within the United States—a developed nation—this rate was also 26% [15], mirroring our own circumstances. This suggests that many factors other than the economic conditions of the country also influence the provision of transitional care. Obstacles to establishing an effective transition program were commonly identified as lack of time, staff and resources [15–17]. However, in the present study and Brazilian survey [10], the most frequently cited barrier was the lack of access to an adult rheumatologist. Correspondingly in the present study, more than half of responders reported that the lack of adult rheumatologists is the main barrier for generating a transition program. In the presented survey, participants overwhelmingly shared a consensus, with over 85% agreement on all aspects of determining transition policies. They emphasized the importance of the transition policies establishing a nationwide transition program and customizing it based on the unique characteristics of healthcare centers and individual patients. A remarkable number of respondents advocated coordinating the transition program with a specific personnel. Furthermore, the designated team member as a transition coordinator was usually a pediatric rheumatologist, according to the current survey. Pediatric rheumatologists generally point to themselves as responsible for the transition process in previous studies [10, 15], while the designated team member as the transition coordinator is usually a specialist nurse in individualized transition programs, including a multidisciplinary team [15]. The insufficiency of auxiliary staff in our country could be the factor leading pediatricians to assume this responsibility. Furthermore, pediatric rheumatology teams often develop strong and enduring connections with their patients, having accompanied them through the various stages of their illnesses [18]. Consequently, they carry a profound responsibility to ensure their patients receive high-quality healthcare as they transition into adulthood.

“Transfer” is typically viewed as a singular moment when responsibility for a patient’s care shifts from a pediatric provider to an adult one. In contrast, “transition” is a comprehensive process that initiates well before the actual transfer moment and extends into young adulthood, encompassing various steps [18]. There is no consensus on the optimal time to start transition preparation in the literature. Different results were obtained from different countries. For instance, Brazilian rheumatologists believed that the transition should not begin before age 15 [10]. Furthermore, there is no consensus on when the transition process will conclude. The rheumatologists in CARRA survey initiated transition discussions with patients and parents between ages 15–17 and completed the transition process after age 19 [15]. In Finland, patients with JIA entered the transition program between ages 16–18, concluding at age 20 [19]. In the presented survey, approximately 70% of participants conducted transitional visits at age 18, while the remainder did so between ages 18–22. Also, a third of respondents completed the transition process at age of 18, another third at ages of 18–20 and the rest between ages of 20–24.

The majority of participants in our survey expressed the opinion that for a successful transition, patients should possess a well-defined strategy encompassing education, career, and disease control. As patients with JIA grow older, they tend to acquire increased knowledge about their condition, exhibit greater independence in managing their health, and develop more explicit vocational plans [20]. In our survey, participants expressed the importance of engaging patients in discussions and providing information about their medical conditions, medications, sexuality, and the risks associated with smoking, alcohol, and narcotics. However, it’s noteworthy that 60% of participants could not discuss career choices due to a lack of time and the attitudes of patients and parents on this issue. Moreover, discussions around sexuality were inhibited by the cultural taboo surrounding this topic in our country, while parents’ reactions hindered conversations regarding tobacco, alcohol, and narcotics use. The presence of psychologists who are familiar with the country’s cultural background in the transition team may provide a receptive environment for addressing these matters with families.

Participants agreed that AYAs should be evaluated with validated questionnaires whether they are ready for transfer or not. However, they argued that the questionnaires should be adapted according to our country’s socioeconomic, cultural, and health insurance conditions. Numerous transfer readiness assessment questionnaires existed, including TRAQ, TRANSITIONQ, and ADAPT [21–23]. Notably, TRAQ, the most commonly used one yielded varying outcomes according to financial status and parental styles among children with rheumatic diseases across countries [24]. There are distinctions between our culture and Western culture, particularly in terms of living arrangements and financial management. In our country, it’s common for children to reside with their parents well into adulthood, typically moving out only upon marriage. Consequently, the responsibility for health insurance and healthcare financing often falls on the shoulders of parents or caregivers. In our culture, parents tend to be highly protective of their children, even as they transition into young adulthood. This protective approach may contribute to a delay in adolescents assuming responsibility for their own health. Therefore, transition care should be prepared to take these cultural differences into consideration, and surveys assessing transition readiness should include these cultural differences. Disease activity constitutes another significant factor in the transition process, as highlighted in a prior study [25]. Ideally, transfers are recommended when the disease is stable. However, in our survey, only one-third indicated that they had a transfer during stable disease. The primary reason limiting transition during stable disease was that certain effective drugs like ustekinumab, secukinumab, and janus kinase inhibitors are not approved in our country for JIA treatment and the restriction of pediatric rheumatologists from prescribing tocilizumab after patients reached 17 years of age [26–29]. Consequently, participants preferred to transfer patients with active diseases requiring these treatments directly to the adult rheumatology department rather than waiting for a stable disease.

Even if there is a well-structured transition program, the transition success in AYAs with rheumatic diseases was below 50%, and 10% of the patients did not continue to follow up after the first adult rheumatology visit [25]. Our participants also stated that patient feedback should be obtained after the first transfer visit to ensure the continuation and adequacy of adult rheumatology follow-up. However, data on the compliance of these patients to follow-up in adult clinics is insufficient.

Our survey bears certain limitations that warrant consideration. Notably, the average duration of professional experience in rheumatology among participants was limited to a mere four years. This constraint is noteworthy given that pediatric rheumatology attained recognition as a subspecialty in 2010, subsequently giving rise to dedicated fellow education programs, with the first practitioners establishing independent clinics post-2013. Furthermore, the responses may not offer a comprehensive representation of the perspectives held by all rheumatologists within our country. Despite the survey being targeted at rheumatology associations, the response was limited to a modest 48 participants. This restriction may be attributed to the large number of patients and intense workload in our country. Additionally, a majority of rheumatologists operate within private clinics. Therefore not being involved in transitional outpatient clinic processes may have reduced the response rate.

## Conclusions

Ensuring that JIA patients receive adequate transition care is imperative. While our country’s current transition care system exhibits certain shortcomings in achieving the ideal transition process, the viewpoints expressed by clinicians align with aspirational standards. Determining the problems is the most important step for creating a standard transition program suitable for the country’s conditions. This survey has furnished insights into the operational dynamics of the transition process within our country. Henceforth, our primary goal is to devise a standard program that comprehensively addresses these inadequacies and establishes the requisite infrastructure to facilitate its seamless execution. The results of this survey clearly showed the necessity of a consensus for transition care. In the second step, a consensus meeting should be arranged to raise the quality of transition care to ideal standards. Consequently, we are hopeful that by consistently working hard and cooperating, we can improve transition care for JIA patients locally and worldwide. We believe that our study will play a valuable role in advancing this important cause.

### Supplementary Information

**Additional file 1.**

## Data Availability

The datasets generated and/or analyzed during the current study are available from the corresponding author on reasonable request.

## Abbreviations

AYAs: Adolescents and young adults
JIA: Juvenile idiopathic arthritis
