# Supplementary material for: Towards a standardized program of transitional care for adolescents with juvenile idiopathic arthritis for Turkey: a national survey study
Source: Pediatr Rheumatol Online J. 2024 Jan 2;22:7. doi: 10.1186/s12969-023-00943-3 (PMC10762787; doi:10.1186/s12969-023-00943-3)
Supplement: Supplementary file 1 — Additional file 1. [file 12969_2023_943_MOESM1_ESM.docx]

Dear participant,

We are currently researching the status of transition programs for juvenile idiopathic arthritis patients within our country. As pediatric and adult rheumatologists, we kindly request your participation in the following survey to gather your insights regarding this matter and the current state of transition programs within your healthcare centers.

**Questionnaire**

1. What is your field of study? a) Pediatric rheumatology b) Adult rheumatology
2. How many years have you studied in the field of rheumatology? (…… years)
3. In which type of center are you studying? a) A university hospital b) A public hospital c) A private hospital
4. Do you have a transition clinic in your center?
5. Yes 2- No
6. If your answer to the 4^th^ question is “no,” please provide the reasons for it.
7. To what extent do you agree with the statement: "There should be a transition program that explains all the steps in the transition process"?
8. Strongly disagree 2- Disagree 3- Neither agree nor disagree 4- Agree 5- Strongly agree
9. To what extent do you agree with the statement: “Transitional care should be individualized to the patient.”

1-Strongly disagree 2- Disagree 3- Neither agree nor disagree 4- Agree 5- Strongly agree

1. Do you individualize the transitional care to the patient?
2. Yes 2- No
3. If your answer to the 8^th^ question is “no,” please provide the reason for it.
4. To what extent do you agree with the statement: “A standard transition program should be generated throughout the country according to the characteristics of the society.”

1-Strongly disagree 2- Disagree 3- Neither agree nor disagree 4- Agree 5- Strongly agree

1. To what extent do you agree with the statement: “A standard transition program should be generated according to characteristics of the centers.”

1-Strongly disagree 2- Disagree 3- Neither agree nor disagree 4- Agree 5- Strongly agree

1. If there is a transition clinic in our center, do you have a standardized transitional program?
2. Yes 2- No
3. If your answer to the 12^th^ question is “yes,” please provide how you determined your transition program.
4. If your answer to the 12^th^ question is “no,” please provide the reason for it.
5. If there is a transition clinic in our center, do you have a multidisciplinary team for the transition?
6. Yes 2- No
7. If your answer to the 15^th^ question is "yes," please explain who the team
8. If your answer to the 15^th^ question is “no,” please provide the reasons for it.
9. To what extent do you agree with the statement: “Team members involved in transitional care should have sufficient interest and knowledge about transitional care.”

1-Strongly disagree 2- Disagree 3- Neither agree nor disagree 4- Agree 5- Strongly agree

1. If there is a transition clinic in our center, do team members in transition care have sufficient interest and knowledge about transition care?
2. Yes 2- No
3. Do you have a person who coordinated the transition period in your center?
4. Yes 2- No
5. If your answer to the 20^th^ question is “yes,” please provide who coordinates the transition period in your center.
6. To what extent do you agree with the statement: "Good collaboration between child and adult health services is necessary."
7. Strongly disagree 2- Disagree 3- Neither agree nor disagree 4- Agree 5- Strongly agree
8. To what extent do you agree with the statement: "Criteria need to be defined for identifying patients eligible for the transition process.”

1-Strongly disagree 2- Disagree 3- Neither agree nor disagree 4- Agree 5- Strongly agree

1. What is the level of collaboration between child and adult health services at your center?

1-Insufficient 2-Poor 3- Adequate 4- Sufficient 5- Perfect

1. Please provide the important factors for you in determining transition time.
2. When (age) preparation of transitional care should be started?
3. 12-14 years old 2- 14-16 years old 3- 16-18 years old
4. Until when (age) should temporary care preparation be continued?

1-up to 14 years old 2-up to 16 years old 3-up to 18 years old

1. Does transition care at your center include a preparation phase?

1-Yes 2- No

1. If your answer to the 28^th^ question is “no,” please provide the reasons for it.
2. To what extent do you agree with the statement: “The data of the transition process should be recorded.”
3. Strongly disagree 2- Disagree 3- Neither agree nor disagree 4- Agree 5- Strongly agree
4. Do you have a regular registration system for the transition process in your center?

1-Yes 2- No

1. If your answer to the 31^st^ question is “no,” please provide the reasons for it.
2. When should the first talk with patients about the transition be made?

1-12-14 years old 2- 14-16 years old 3- 16-18 years old

1. To what extent do you agree with the statement: “The patients' readiness for the transition should be tested before starting the transition outpatient clinic.”

1- Strongly disagree 2- Disagree 3- Neither agree nor disagree 4- Agree 5- Strongly agree

1. Would you test the patients' readiness before starting the transition outpatient clinic?
2. Yes 2- No
3. If your answer to the 35^th^ question is “no,” please provide the reasons for it.
4. To what extent do you agree with the statement: “The timing of transition steps should be decided together with parents.”
5. Strongly disagree 2- Disagree 3- Neither agree nor disagree 4- Agree 5- Strongly agree
6. Do you involve parents in the decision when timing transition steps in their clinics?
7. Yes 2- No
8. If your answer to the 38^th^ question is “no,” please provide the reasons for it.
9. To what extent do you agree with the statement: “The timing of all transition steps until age 18 should be cleared during the transition planning steps.

1-Strongly disagree 2- Disagree 3- Neither agree nor disagree 4- Agree 5- Strongly agree

1. To what extent do you agree with the statement: “During the transition readiness step, parents' new changing roles in the transition process should be discussed.”

1-Strongly disagree 2- Disagree 3- Neither agree nor disagree 4- Agree 5- Strongly agree

1. To what extent do you agree with the statement: “The patients should be encouraged to answer questions about their illness, treatment, pain, education, and activities during visits after the age of 12.”

1-Strongly disagree 2- Disagree 3- Neither agree nor disagree 4- Agree 5- Strongly agree

1. Can patients aged 14 to 16 briefly describe their illness?

1-Yes 2- No

1. If your answer to the 41^st^ question is “no,” please provide the reasons for it.
2. Can your patients aged 14 to 16 briefly describe their medications, detailing their usage and the reasons behind them?

1-Yes 2- No

1. If your answer to the 43^rd^ question is “no,” please provide the reasons for it.
2. Do you inform the patients about career choices related to illness?

1-Yes 2- No

1. If your answer to the 45^th^ question is “no,” please provide the reasons for it.
2. To what extent do you agree with the statement: “The patients should be informed about sexuality and the harms of smoking, alcohol, and narcotics.”

1-Strongly disagree 2- Disagree 3- Neither agree nor disagree 4- Agree 5- Strongly agree

1. Do you inform the patients about sexuality and the harms of smoking, alcohol, and narcotics?

1-Yes 2- No

1. If your answer to the 48^th^ question is “no,” please provide the reasons for it.
2. To what extent do you agree with the statement: “The patients should be informed about the effects of the disease and treatment on fertility and pregnancy between the ages of 14-16.”

Strongly disagree 2- Disagree 3- Neither agree nor disagree 4- Agree 5- Strongly agree

1. Do you inform the patients about the effects of the disease and treatment on fertility and pregnancy between the ages of 14-16.”

1-Yes 2- No

1. If your answer to the 51^st^ question is “no,” please provide the reasons for it.
2. When should the transition program start?

1-12-14 years old, 2- 14-16 years old, 3- 16-18 years old, 4- 18 years old

1. To what extent do you agree with the statement: “The transfer center should be determined jointly by the family, patient, and clinician.”

1-Strongly disagree 2- Disagree 3- Neither agree nor disagree 4- Agree 5- Strongly agree

1. Do you select the transfer center jointly by the family, patient, and clinician?

1-Yes 2- No

1. If your answer to the 55^th^ question is “no,” please provide the reasons for it.
2. To what extent do you agree with the statement: “Transfer readiness should be assessed through validated questionnaires such as TRAQ and transition Q.”

1-Strongly disagree 2- Disagree 3- Neither agree nor disagree 4- Agree 5- Strongly agree

1. Do you think these questionnaires should be rearranged?

1-Yes 2- No

1. If your answer to the 58^th^ question is “yes,” please specify in what respect it should be rearranged.
2. To what extent do you agree with the statement: “Transition visits should be done between 18 and 20 years.”

1-Strongly disagree 2- Disagree 3- Neither agree nor disagree 4- Agree 5- Strongly agree

1. When do you schedule the transition visits in your center?

1-18 years old 2- 18-20 years old 3- 20-22 years old

1. To what extent do you agree with the statement: “The time of transition visits should be specified jointly with patients and parents.”

1-Strongly disagree 2- Disagree 3- Neither agree nor disagree 4- Agree 5- Strongly agree

1. Do you specify the time of transition visits jointly with patients and parents?
2. Yes 2- No
3. To what extent do you agree with the statement: “The transfer should be timed at stable disease.”

1-Strongly disagree 2- Disagree 3- Neither agree nor disagree 4- Agree 5- Strongly agree

1. Do you transfer the patients to adult rheumatology care at stable disease?

1-Yes 2- No

1. If your answer to the 65^th^ question is “no,” please provide the reasons for it.
2. To what extent do you agree with the statement: “The transfer letter, the registry monitoring the transition process, and the epicrisis should be forwarded to the adult rheumatology team.”

1-Strongly disagree 2- Disagree 3- Neither agree nor disagree 4- Agree 5- Strongly agree

1. Do you forward the transfer letter, the registry monitoring the transition process, and the epicrisis to the adult rheumatology team?

1-Yes 2- No

1. If your answer to the 68^th^ question is “no,” please provide the reasons for it.
2. To what extent do you agree with the statement: “The transfer visit should be conducted in an environment suitable for AYAs.”

1-Strongly disagree 2- Disagree 3- Neither agree nor disagree 4- Agree 5- Strongly agree

1. Do you have a suitable environment for AYAs during the transfer visit?

1-Yes 2- No

1. If your answer to the 71^st^ question is “no,” please provide the reasons for it.
2. To what extent do you agree with the statement: “At least one transfer visit should be done together with adult rheumatology and pediatric rheumatology.”

1-Strongly disagree 2- Disagree 3- Neither agree nor disagree 4- Agree 5- Strongly agree

1. To what extent do you agree with the statement: “Feedback (maybe online) should be provided to the pediatric rheumatology team from the patient (and parent if necessary) about the first visit in adult care.”

1-Strongly disagree 2- Disagree 3- Neither agree nor disagree 4- Agree 5- Strongly agree

1. Do you take feedback from the patient (and parent if necessary) about the first visit in adult care?

1-Yes 2- No

1. If your answer to the 75^th^ question is “no,” please provide the reasons for it.
2. To what extent do you agree with the statement: “The second visit should be made together with pediatric and adult rheumatologists in the adult rheumatology outpatient clinic.”

1-Strongly disagree 2- Disagree 3- Neither agree nor disagree 4- Agree 5- Strongly agree

1. To what extent do you agree with the statement: “Annual visits to the pediatric rheumatology clinic should be recommended for patients and their parents until the age of 24 to ensure the continuation of follow-up appointments.”

1-Strongly disagree 2- Disagree 3- Neither agree nor disagree 4- Agree 5- Strongly agree

1. To what extent do you agree with the statement: “The last visit should be made with only the adult rheumatologist in the adult rheumatology clinic.”

1-Strongly disagree 2- Disagree 3- Neither agree nor disagree 4- Agree 5- Strongly agree

1. To what extent do you agree with the statement: “The patient should be seen alone in the visits after the transfer visit.”

1-Strongly disagree 2- Disagree 3- Neither agree nor disagree 4- Agree 5- Strongly agree

1. To what extent do you agree with the statement: “Before completing the transition process, it should be sure the patient can schedule follow-up appointments.”

1-Strongly disagree 2- Disagree 3- Neither agree nor disagree 4- Agree 5- Strongly agree

1. To what extent do you agree with the statement: “The transition process should be completed in 20-24 years”.

1-Strongly disagree 2- Disagree 3- Neither agree nor disagree 4- Agree 5- Strongly agree

1. When do you complete the transition process?

1-18 years old 2- 18-20 years old 3- 20-24 years old

1. To what extent do you agree with the statement: “Patients should have a clear plan for education, professional life, and disease management before completing the transition.”

1-Strongly disagree 2- Disagree 3- Neither agree nor disagree 4- Agree 5- Strongly agree
